# Whole Body Bone Tissue and Cardiovascular Risk in Rheumatoid Arthritis

**DOI:** 10.1155/2014/465987

**Published:** 2014-04-08

**Authors:** Claudiu Popescu, Violeta Bojincă, Daniela Opriş, Ruxandra Ionescu

**Affiliations:** ^1^“Sfânta Maria” Clinical Hospital, 37-39 Ion Mihalache Boulevard, District 1, 011192 Bucharest, Romania; ^2^Department of Internal Medicine and Rheumatology, “Carol Davila” University of Medicine and Pharmacy, 011192 Bucharest, Romania

## Abstract

*Introduction*. Atherosclerosis and osteoporosis share an age-independent bidirectional correlation. Rheumatoid arthritis (RA) represents a risk factor for both conditions. *Objectives*. The study aims to evaluate the connection between the estimated cardiovascular risk (CVR) and the loss of bone tissue in RA patients. *Methods*. The study has a prospective cross-sectional design and it includes female in-patients with RA or without autoimmune diseases; bone tissue was measured using whole body dual X-ray absorptiometry (wbDXA); CVR was estimated using SCORE charts and PROCAM applications. *Results*. There were 75 RA women and 66 normal women of similar age. The wbDXA bone indices correlate significantly, negatively, and age-independently with the estimated CVR. The whole body bone percent (wbBP) was a significant predictor of estimated CVR, explaining 26% of SCORE variation along with low density lipoprotein (*P* < 0.001) and 49.7% of PROCAM variation along with glycemia and menopause duration (*P* < 0.001). Although obese patients had less bone relative to body composition (wbBP), in terms of quantity their bone content was significantly higher than that of nonobese patients. *Conclusions*. Female patients with RA and female patients with cardiovascular morbidity have a lower whole body bone percent. Obese female individuals have higher whole body bone mass than nonobese patients.

## 1. Introduction 

Atherosclerosis and osteoporosis share an age-independent bidirectional correlation [1, 2], although they were considered to be independent pathologies. On one hand, low bone mass is associated with subclinical [3] and clinical atherosclerosis, manifested as cardiovascular morbidity [4, 5] and mortality [6]. On the other hand, cardiovascular disease is associated with low bone mass and with a higher risk of fragility fractures [7]. There is no unifying theory which can explain a deterministic link between atherosclerosis and osteoporosis, if any. Certainly, bone modeling and vascular calcification have common biological processes and risk factors (e.g., smoking, inactivity, and inflammation) [8]. We can cite RA among these factors, as a crossroad of atherosclerosis and osteoporosis. The main cause of mortality in RA is the cardiovascular pathology [9, 10]. Adding to the classical cardiovascular risk (CVR) factor, chronic inflammation [11, 12] and disease duration [12] contribute decisively to the excess cardiovascular mortality in RA. The background of this observation is the inflammatory pathogenesis of RA which is associated with accelerated atherosclerosis [13], both clinically and subclinically [14, 15]. Therefore, EULAR recommends annual CVR evaluation in RA patients [16], knowing the fact that RA is an independent CVR factor [9], similar in importance with diabetes mellitus (DM) [17]. One of the main consequences of RA is osteoporosis. It appears early in the disease course, two times more prevalent than in the general population [18, 19]. Adding to the risk factor of primary osteoporosis, the bone loss in RA is associated with the presence of autoantibodies [18], glucocorticoid therapy, disease activity (the activation of osteoclasts by mean of the RANK pathway) [20, 21], and disability. Because of the high risk of fractures in RA [22, 23], RA patients should routinely undergo dual X-ray absorptiometry (DXA) in order to diagnose and monitor osteoporosis. The advantage is that at the same time with DXA measurements one can evaluate the patient's body composition. DXA has been validated as a precise method for estimating body composition [24]. If coupled with CVR estimation, the DXA technique could become the common denominator for the complex management of atherosclerosis and osteoporosis in RA. In this context, the study aims to evaluate the correlation between the estimated CVR and whole body bone loss, emphasizing RA as a common risk factor for both conditions.

## 2. Methods and Materials

### 2.1. Patients

The study was cross-sectionally designed to include female in-patients, admitted to the hospital in the random order of presentation for clinical and biological reevaluation. The subjects were classified as either RA patients, according to the 2010 ACR/EULAR criteria [25], either as having no chronic inflammatory autoimmune disease. Each patient gave written informed consent and the study was approved by the local ethics committee.

### 2.2. Measurements

**Figure 1:**
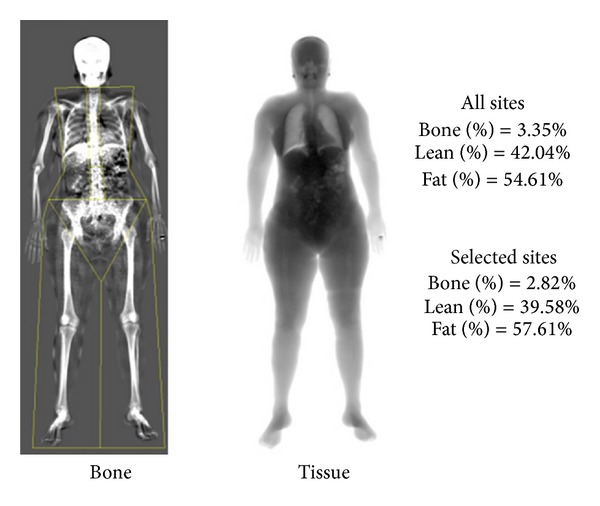
Body mass composition result, indicating the bone tissue percent with regard to body mass (Lexxos C05LX223 software v6.0551).

Demographic data and smoking status were recorded by means of anamnesis. The bone tissue was evaluated by a single certified clinical densitometrist (CP) using whole body DXA (wbDXA; Lexxos C05LX223), which records variables such as bone tissue density/mass (BTD/M), *T*- and *Z*-scores (*T*/*Z*_wb_), and bone tissue percent (BTP; Figure 1). The classic anthropometric indices, such as height, weight, abdominal circumference (AC), and hip circumference (HC), were measured using a mechanical scale (0.1 kg maximal error), a wall stadiometer (0.3 cm maximal error), and a centimeter graded tape. Using these measurements, we computed the derived anthropometric indices: body mass index (BMI; weight divided by square height), waist-to-hip ratio (WHR; AC divided by HC), conicity index (CI; AC divided by the square root of weight to height ratio multiplied by 0.109). Erythrocyte sedimentation rate (ESR) was measured by the Westergren method (normal range according to age). The concentration of C-reactive protein (CRP) was measured by immunonephelometry (normal range < 5 mg/L). Arterial hypertension (AHT) was defined after two measurements (auscultatory sphygmomanometer; 5 mmHg error) if systolic pressure ≥ 140 mmHg, or diastolic pressure ≥ 90 mmHg, or if the patient was on blood pressure lowering therapy [26]. Ischemic heart disease (IHD) was defined on electrocardiographic criteria or on a history of acute coronary syndromes, stable angina, conduction and rhythm disturbances, and ischemic heart failure [27]. The recorded antiplatelet agents were aspirin and clopidogrel. Dyslipidemia was defined as triglycerides > 150 mg/dL, total cholesterol > 200 mg/dL, low-density lipoproteins (LDL) > 100 mg/dL, high-density lipoproteins (HDL) < 50 mg/dL, or treatment with statins and fibrates [28]. Diabetes mellitus was defined as two fasting plasma glucose levels > 126 mg/dL (FPG), one FPG > 200 mg/dL, or treatment with insulin/oral antidiabetic agents [29]. Obesity was defined as a BMI ≥ 30 kg/m^2^. The metabolic syndrome was defined using the 2006 International Diabetes Federation criteria [30]. The CVR was estimated using high risk SCORE charts, appropriate for the Romanian population [31], and quick check and health check PROCAM applications [32], according to EULAR recommendations [16].

### 2.3. Statistics

The normal distribution of data was assessed using descriptive statistics, normality and stem-and-leaf plots, and the Lilliefors corrected Kolmogorov-Smirnov test. Normally distributed data were reported as means with standard deviations and their correlations and differences were assessed with Pearson coefficients and* t*-tests, respectively. Non normally distributed data were reported as medians with intervals and their correlations and differences were assessed with Spearman coefficients and Mann Whitney tests, respectively. Qualitative data were expressed as absolute and percent frequency and their differences were assessed using *χ* ^2^ tests (or Fisher tests where appropriate). The influence of whole body bone tissue on CVR was studied using multiple linear regression and covariance analysis (ANCOVA). All tests were considered significant if *P* < 0.05 and were done using SPSS v.17 for Windows (SPSS Inc., Chicago, USA, 2008).

## 3. Results

### 3.1. RA-Normal Subjects Comparison

Table 1 summarizes the variables recorded in the two groups. Although RA patients do not have longer menopause duration or higher menopause frequency, most of their wbDXA indices are significantly lower than those of normal patients. It should be noted that RA patients had a mean disease duration of 12.8 ± 10 years, 66 patients (86.8%) were RF positive and 44 patients (57.9%) were anti-CCP positive. RA was moderately active in the studied group (mean DAS28 of 4.25 ± 1.21; mean SDAI of 63.3 ± 40.9). As to treatment regimes, 68 patients (89.5%) were receiving methotrexate, 35 patients (46%) were receiving oral glucocorticoids, and 25 patients (32.9%) were receiving bisphosphonates.

### 3.2. Bone Tissue and Cardiovascular Risk

As pointed out in Table 2, the wbDXA indices of bone tissue are significantly and negatively correlated with the CVR estimation by all three instruments. The covariance analysis revealed that this correlation is independent of age, menopause, IHD, smoking, BMI, obesity, AHT, dyslipidemia, DM, inflammation, and metabolic syndrome.

The multiple linear regression of BTP with CVR scores revealed notable results. Except for BTP, SCORE correlated significantly with age, menopause duration, height, AC, HC, WHR, BMI, CI, LDL, and total cholesterol. Of these, we excluded age, since it is part of SCORE protocol, and WHR, BMI, and CI, since they where derived from variables that were already correlated with SCORE. The remaining variables significantly explained 47% of SCORE variation (*R* ^2^ = 0.472; *P* < 0.001; standard method), but the contribution of height, AC, HC, and total cholesterol were not significant, being thus eliminated from the regression model. The two remaining predictors were introduced in the equation in the order LDL-BTP and significantly explained 26% of SCORE variation (*R* ^2^ = 0.262; *P* < 0.001; hierarchical method). In this two-parameter model, LDL independently explained 3.4% of SCORE variation, while BTP explained 25%.

Except for BTP, PROCAMqc correlated significantly with age, menopause duration, height, body mass, AC, HC, WHR, BMI, CI, and glycemia. Of these, we excluded height, body mass, WHR, BMI, and CI on the same grounds. The standard multiple linear regression of the remaining variables with PROCAMqc generated a significant equation which predicted 51.3% of its variation (*R* ^2^ = 0.513; *P* < 0.001). The contribution of AC and HC was not significant though. The three remaining predictors (menopause duration, glycemia, and BTP) were introduced in the equation in that order and significantly explained 50% of PROCAMqc variation (*R* ^2^ = 0.497; *P* < 0.001; stepwise method). In this three-parameter model, menopause duration independently explained 23.3% of PROCAMqc variation; glycemia explained 16.6%, while BTP explained 10%.

### 3.3. Bone Tissue and CVR Factors

As was expected from bone measurements, the wbDXA indices correlated significantly and negatively with age and were significantly higher in the premenopause period (Table 3). Moreover, these indices correlated significantly with the classical anthropometric measurements, in an age-independent manner (Table 4). The wbDXA bone indices varied significantly according to the presence or the absence of classic CVR factors (Table 5). For example, obese patients had lower BTP than nonobese patients, but their bone quantity was significantly higher. It is interesting that patients with high fasting glycemia, when compared with normal glycemic patients, behaved in the same manner as obese patients with regard to wbDXA bone indices. In general, patients with inflammatory and cardiovascular pathology had a lower BTP.

## 4. Discussion

In clinical practice, bone density regions of interest in adults include the lumbar spine, the femoral neck, and the distal radius, since these measurements have a higher predicting value in the recognition of osteoporosis than the whole body measurements. Since the purpose our study was not to diagnose osteoporosis, but instead to evaluate the link between bone loss and cardiovascular risk, a whole body approach was deemed more appropriate in this fundamental science query.

The correlation between cardiovascular pathology and bone tissue has been observed in the general population, but RA is an appropriate condition in which the two entities can be studied. As to bone tissue, the previous studies noted that RA patients, compared to normal individuals, have a lower DXA bone density in the regions used for the diagnosis of osteoporosis (lumbar spine, hip, and distal radius) [33]. Our study proves that the entire skeleton of RA patients exhibits lower bone density and lower bone mass than normal individuals. It seems that inflammation, glucocorticoid treatment, and low BMI are accounting for this difference, since the two groups did not differ significantly in terms of the other osteoporosis risk factor included in the study (age, menopause prevalence and duration, and smoking—see Table 1). As to atherosclerosis, RA patients had a higher CVR than normal individuals, in spite of the fact that the control group did not have a favorable cardiovascular profile: the controls were non-RA but were admitted to the hospital for different internal medicine diagnoses. This is why the two groups had similar prevalence of metabolic syndrome, AHT, IHD, and DM and had a higher prevalence of obesity.

Without suggesting any deterministic direction, our study reports a correlation between wbDXA bone indices with the CVR estimated on three different scales (Table 2). This correlation is real within the study's limitations, since it does not depend on the effect of the established CVR factors (age, menopause, smoking, obesity, AHT, dyslipidemia, inflammation, DM, and metabolic syndrome). We must emphasize that all types of wbDXA bone measurement showed independent correlations with CVR, both those that estimate absolute bone mass (g) and those that measure aria density (g/cm^2^) and body mass fraction (%). Secondly, these correlations are negative, meaning that CVR increases as bone mass, density, and percent decreases. Of all the wbDXA bone indices, BTP had the strongest correlations with CVR. For this reason it was used in regression models, proving to be an independent and significant predictor of CVR estimation, alongside LDL, glycemia, and menopause duration.

The correlation of wbDXA bone indices with CVR exceeds the scope of the clinical instruments used to estimate it (SCORE, PROCAM), which do not incorporate important variables such as the presence or the absence of the metabolic syndrome. Our data showed that individuals with cardiovascular morbidity are different (see Table 5). Three comments are pertinent at this stage. The first refers to the controversial relationship between osteoporosis and obesity. One can argue that BMI-defined obesity protects from osteoporosis, most likely by means of adipocyte estrogen production [34, 35]. This protective effect is not warranted though [36]. Our data offer an extra argument in favor of obesity's antiosteoporotic effect: patients with BMI > 30 kg/m^2^ have a higher whole body bone mass and a lower bone percent than patients with BMI < 30 kg/m^2^. However, if we define obesity by body fat percent, this observation reverses. This apparent contradiction, noted also by Migliaccio et al. [35], resides in the definition of bone percent, defined as bone mass divided by body mass: BMI-defined obese individuals have a slightly higher bone mass than nonobese individuals, but their fat mass is disproportionally higher, enough to produce a smaller bone percent. This explains the difference in BTP, but why do BMI-defined obese individuals have more bone? Our data indicate that BMI-defined obese and nonobese patients have similar height (159.4 cm; *P* > 0.1) and similar bone density. Therefore, BMI-defined obese individuals must have thicker and/or wider bones.

The second comment refers to the unusual behavior with regard to wbDXA bone indices when the study population sample was divided in two subgroups according to glycemia values: hyperglycemic patients behaved exactly like obese patients. It is possible that this effect is the consequence of the significantly higher obesity prevalence among hyperglycemic patients (50%; 34/68) than in normal glycemic patients (20.5%; 15/73; *P* < 0.001).

Lastly, we must mention the effect of inflammation on wbDXA bone indices, namely, that patients with elevated inflammatory markers had less bone in terms of density and body percent. Moreover, inflammation markers correlated significantly with the anthropometric predictors of cardiovascular morbidity and mortality: ESR correlated positively with AC (*r* = 0.175; *P* = 0.038) and HC (*r* = 0.184; *P* = 0.029) and negatively with HDL (*r* = −0.258; *P* = 0.002), while CRP correlated positively with body mass (*r* = 0.215; *P* = 0.011), AC (*r* = 0.258; *P* = 0.002), HC (*r* = 0.278; *P* = 0.001), BMI (*r* = 0.242; *P* = 0.004), and CI (*r* = 0.237; *P* = 0.005) and negatively with HDL (*r* = −0.391; *P* < 0.001). These observations strengthen the evidence that prove the involvement of inflammation in osteoporosis and atherosclerosis, so well exemplified in RA, but also raises the question of how anthropometry influences the production of inflammatory markers. Integrating these results one must bear in mind study limitations, namely, the cross-sectional design, which did not allow follow-up of these patients, the lack of bone markers measurements, and the use of a surrogate cardiovascular morbidity and mortality marker.

## 5. Conclusion

From a deterministic point of view on atherosclerosis-bone tissue, we noted that on one hand RA patients have significantly lower whole body bone tissue indices and a significantly higher cardiovascular risk compared to normal subject, and that on the other hand inflammation is associated with lower whole body bone tissue indices and is correlated with anthropometric and nonanthropometric cardiovascular risk predictors. These wbDXA bone indices correlate significantly, independently, and negatively with CVR estimation on three different clinical scales. Of all bone measurements, whole body bone percent is the best predictor of CVR, which is the reason for its possible clinical application. The connection between atherosclerosis and osteoporosis is not limited only to the correlation with CVR estimates, but we observed that patients with cardiovascular morbidity (IHD, metabolic syndrome, DM, AHT, and dyslipidemia) had lower wbDXA bone indices. Another established CVR factor, BMI-defined obesity, is associated with a higher whole body bone mass. This bone tissue surplus in obese patients was not accounted by whole body bone density nor by height; therefore it is probably explained by higher individual bone thickness and/or width.

## Figures and Tables

**Table 1:** Comparison between RA and normal patients.

	Variable	Normal (*n* = 66)	RA (*n* = 75)	*P*
	Age (years)	56.7 (9.7)	56 (11.4)	ns
	Menopause	53 (80.3%)	57 (76%)	ns
	MPD (years)	13.1 (9)	14.3 (7.9)	ns

Bone tissue	wbBTD (g/cm^2^)	0.797 (0.093)	0.763 (0.091)	0.032
	wbBTM (kg)	1.56 (0.16–2.14)	1.50 (0.82–2.03)	0.028*
	*Z*_wb_ (sd)	−0.75 (−2.5–1.5)	−0.91 (−3.8–0.9)	0.015*
	*T*_wb_ (sd)	−1.1 (1)	−1.5 (1)	0.035
	BTP (%)	2.76 (0.38)	2.73 (0.37)	ns
	BTD_lumbar_ (g/cm^2^)	0.887 (0.159)	0.857 (0.212)	ns
	*T*_lumbar_ (sd)	−0.5 (−3.7–2.8)	−0.7 (−4.4–2.5)	ns*

Anthropometrics	Height (cm)	159 (5.7)	160 (6.2)	ns
	Body mass (kg)	74.5 (13.4)	69.8 (14)	0.044
	WC (cm)	95.3 (13.6)	94.3 (12)	ns
	HC (cm)	107.9 (11.1)	107.1 (11)	ns
	WHR	0.88 (0.06)	0.88 (0.05)	ns
	BMI (kg/m^2^)	29.5 (4.9)	27.3 (4.9)	0.007
	CI	0.60 (0.14)	0.57 (0.12)	ns

Laboratory	LDL (mg/dL)	126.5 (38.3)	131.5 (30.2)	ns
	HDL (mg/dL)	58.5 (19.8)	52.2 (11.3)	0.021
	TC (mg/dL)	213.7 (48)	208.4 (41.1)	ns
	TG (mg/dL)	110.5 (43–843)	107.5 (43–258)	ns*
	FPG (mg/dL)	103.5 (80–210)	96 (74–243)	0.001*
	ESR (mm/h)	20 (3–126)	28 (4–103)	0.008*
	CRP (mg/L)	3.5 (0.2–155)	6.6 (0.4–183)	0.024*

Cardiovascular risk	AHT	42 (63.6%)	37 (49.3%)	ns
	Anti-AHT treatment	38 (57.6%)	43 (57.3%)	ns
	IHD	20 (30.3%)	29 (38.7%)	ns
	Antiplatelet	17 (25.8%)	25 (33.3%)	ns
	Dyslipidemia	49 (74.2%)	67 (89.3%)	0.019
	Statins/fibrates	15 (22.7%)	26 (34.7%)	ns
	DM	10 (15.2%)	3 (4%)	0.022
	MetS	41 (62.1%)	45 (60%)	ns
	Obesity	31 (47%)	18 (24%)	0.004
	Inflammation	24 (36.4%)	49 (65.3%)	0.001
	Smoking	16 (24.2%)	7 (9.3%)	0.017
	PROCAMqc (%)	2.1 (1.4)	2.7 (1.9)	0.033

*Mann-Whitney test; RA: rheumatoid arthritis; MPD: menopause duration; BT M/D/P: bone tissue mass/density/percent; WC: waist circumference; HC: hip circumference; WHR: waist to hip ratio; BMI: body mass index; CI: conicity index; L/H DL: low/high density lipoproteins; TC: total cholesterol; TG: triglycerides; FPG: fasting plasma glucose; ESR: erythrocyte sedimentation rate; CRP: C-reactive protein; AHT: arterial hypertension; IHD: ischemic heart disease; DM: diabetes mellitus; MetS: metabolic syndrome; wb: whole body; qc: quick check; ns: not significant; sd: standard deviation.

**Table 2:** Correlation between wbDXA bone indices and estimated CVR.

	RA (n = 75)		Normal (n = 66)		All (n = 141)^&^	
	*r*	*P*	*r*	*P*	*r*	*P*
PROCAMqc						
wbBTD	−0.262	0.024	−0.275	0.026	−0.415	<0.001^§^
*T*_wb_	−0.256	0.028	−0.278	0.024	−0.419	<0.001^§^
BTP	−0.645	<0.001^§^	−0.634	<0.001^§^	−0.549	<0.001^§^
BTD_lumbar_	ns	ns	0.243	0.049	ns	ns
*T*_lumbar_	ns	ns	0.243	0.049	ns	ns
PROCAMhc						
wbBTD	−0.311	<0.001^§^	−0.261	0.034	−0.311	<0.001^§^
*T*_wb_	−0.307	<0.001^§^	−0.261	0.034	−0.307	<0.001^§^
BTP	−0.615	<0.001^§^	−0.592	<0.001^§^	−0.615	<0.001^§^
BTD_lumbar_	−0.323	0.006^§^	ns	ns	−0.282	0.001
*T*_lumbar_	−0.322	0.006^§^	ns	ns	−0.283	0.001
SCORE*						
wbBTD	−0.413	<0.001^§^	−0.430	<0.001^§^	−0.437	<0.001^§^
wbBTM	−0.269	0.001	ns	ns	−0.285	0.001^§^
*T*_wb_	−0.417	<0.001	−0.437	<0.001	−0.441	<0.001^§^
BTP	−0.550	<0.001^§^	−0.525	<0.001^§^	−0.525	<0.001^§^

*Spearman coefficients, the rest being Pearson coefficients.

^§^correlations that remain significant after controlling for age.

^&^correlations made by controlling for RA diagnosis.

CVR: cardiovascular risk; ns: not significant; wb: whole body.

**Table 3:** Whole body bone tissue according to age and menopause (*n* = 141).

	Age		MPD		Before menopause	After menopause	*P*
	*r*	*P*	*r*	*P*	(n = 31)	(n = 110)	
wbBTD (g/cm^2^)	−0.349	<0.001	−0.374	<0.001	0.828 (0.065)	0.765 (0.095)	<0.001
wbBTM (kg)	−0.204	0.016	−0.320	0.001	1.59 (0.26)	1.49 (0.26)	ns
*T*_wb_ (sd)	−0.351	<0.001	−0.368	<0.001	−0.78 (0.72)	−1.45 (1.05)	<0.001
BTP (%)	−0.526	<0.001	−0.226	0.017	3.1 (0.4)	2.6 (0.3)	<0.001

Pearson correlations controlling for RA.

*T*_lumbar_, BTD_lumbar_, and *Z*_wb_ had no significant correlations/differences.

MPD: menopause duration; sd: standard deviations; ns: not significant; wb: whole body.

**Table 4:** Correlation of wbDXA bone indices and anthropometric indices (*n* = 141).

		*H *	*M *	AC	HC	WHR	BMI	CI
wbBTD	*r*	0.231	0.320	0.209	0.238	ns	0.275	0.257
	*P*	0.001	<0.001	0.014	0.005	ns	0.001	0.002

wbBTM	*r*	0.546	0.639	0.440	0.529	ns	0.493	0.482
	*P*	<0.001	<0.001	<0.001	<0.001	ns	<0.001	<0.001

*Z*_wb_	*r*	0.192	0.266	ns	0.181	ns	0.232	0.215
	*P*	0.024	0.002	ns	0.033	ns	0.006	0.011

*T*_wb_	*r*	0.209	0.330	0.213	0.242	ns	0.296	0.267
	*P*	0.011	<0.001	0.012	0.004	ns	<0.001	0.001

BTP	*r*	ns	−0.660	−0.713	−0.718	−0.325	−0.683	−0.671
	*P*	ns	<0.001	<0.001	<0.001	<0.001	<0.001	<0.001

BTD_lumbar_	*r*	ns	ns	ns	ns	0.195	ns	ns
	*P*	ns	ns	ns	ns	0.021	ns	ns

*T*_lumbar_	*r*	ns	ns	ns	ns	0.195	ns	ns
	*P*	ns	ns	ns	ns	0.021	ns	ns

Bivariate partial correlations controlling for age and RA.

Sd: standard deviation, H: height, M: body mass; ns: not significant.

**Table 5:** wbDXA bone indices according to cardiovascular morbidity (*n* = 141).

	Nonobese (*n* = 92)	Obese (*n* = 49)	*P*	Nfpg (*n* = 73)	HFPG (*n* = 68)	*P*
wbBTD (g/cm^2^)	0.771 (0.091)	0.795 (0.096)	ns	0.762 (0.095)	0.798 (0.088)	0.022
wbBTM (kg)	1.46 (0.21)	1.63 (0.30)	<0.001	1.46 (0.22)	1.57 (0.28)	0.012
*Z*_wb_ (sd)	−1.06 (0.84)	−0.65 (0.99)	0.011	−1.16 (0.84)	−0.65 (0.92)	0.001
*T*_wb_ (sd)	−1.4 (0.99)	−1.1 (1)	ns	−1.51 (1)	−1.10 (0.9)	0.017
BTP (%)	2.9 (0.3)	2.5 (0.3)	<0.011	2.9 (0.3)	2.5 (0.3)	ns

	No IS (n = 68)	With IS (n = 73)	P	No IHD (n = 62)	With IHD (n = 79)	P

BTP (%)	2.8 (0.4)	2.6 (0.4)	0.018	2.8 (0.4)	2.6 (0.2)	0.004
BTD_lumbar_ (g/cm^2^)	0.907 (0.164)	0.838 (0.205)	0.030	0.878 (0.186)	0.858 (0.196)	ns
T_lumbar_ (sd)	−0.28 (1.2)	−0.79 (1.5)	0.030	−0.49 (1.3)	−0.63 (1.4)	ns

	No MS (n = 55)	With MS (n = 86)	P	No DM (n = 128)	With DM (n = 13)	P

BTP (%)	2.9 (0.4)	2.6 (0.3)	<0.001	2.8 (0.4)	2.4 (0.3)	0.001

	No DL (n = 25)	With DL (n = 116)	P	No AHT (n = 62)	With AHT (n = 79)	P

BTP (%)	2.9 (0.4)	2.7 (0.3)	0.004	2.9 (0.4)	2.6 (0.3)	<0.001

Sd: standard deviation; ns: not significant; n/Hfpt: normal/high plasma fasting glucose; IS: inflammatory syndrome; DL: dyslipidemia; MS: metabolic syndrome.
